# Cartilage Stiffness Effect on Foot Biomechanics of Chinese Bound Foot: A Finite Element Analysis

**DOI:** 10.3389/fphys.2018.01434

**Published:** 2018-10-11

**Authors:** Yan Zhang, Jan Awrejcewicz, Julien S. Baker, Yaodong Gu

**Affiliations:** ^1^Faculty of Sports Science, Ningbo University, Ningbo, China; ^2^Department of Automation, Biomechanics and Mechatronics, Lodz University of Technology, Lódź, Poland; ^3^Institute for Clinical Exercise and Health Science, University of the West of Scotland, Paisley, United Kingdom

**Keywords:** biomechanics, gait, human locomotion, bound foot, finite element analysis

## Abstract

The purpose of this study is to investigate the effect of cartilage stiffness on inner foot biomechanics of Chinese bound foot while balanced standing using finite element method. A three-dimensional FE model of bound foot involving 28 bones, 72 ligaments, 5 plantar fascia, cartilages, and encapsulated soft tissue was constructed and validated. To conduct the sensitivity analysis of cartilage stiffness, the incremental Young’s modulus of 1, 5, 10, and 15 MPa were assigned to the cartilage. 25% of the body weight was applied to the Achilles tendon to adjust the anterior- posterior displacement of center of pressure agreeable with the measured result. As the Young’s modulus of cartilage increased, the peak von Mises stress in the fifth metatarsal increased obviously, while that in the calcaneus remains unchanged. The plantar fascia experienced reduced total tension with stiffer cartilage. The cartilage stiffening also caused a general increase of contact pressure at mid- and forefoot joints. Cartilage stiffening due to foot binding gave rise to risks of foot pain and longitude arch damage. Knowledge of this study contributes to the understanding of bound foot biomechanical behavior and demonstrating the mechanism of long-term injury and function damage in terms of weight-bearing due to foot binding.

## Introduction

The human foot is a complex mechanical structure consisting of 28 bones, a number of ligaments, muscles, and other connective tissues. As the terminal portion of limb, it bears body weight and allows locomotion. Regardless of causes, the bone or joint deformity may cause functional impairments (Rao et al., 2012). Foot binding, an unique Chinese custom, had ever gained great popularity and enduring influence on Chinese history. For over 1000 years, young girls about 4- to 7-years-old curled (folded) their second to fifth phalanges under the sole wrapped tightly with bandage in order to compress the foot. In addition to phalange fracture, the metatarsals were rearranged into an extremely high arch (Zhang et al., 2014) and the calcaneus was reoriented sagittally toward the alignment of the long axis of the lower leg (Howard and Pillinger, 2010). Thus, a smaller and narrower foot size forms, which was considered to be a means of displaying social status and was correspondingly adopted as a symbol of beauty in ancient Chinese culture (Greenhalgh, 1977). The bound foot was also believed as the object of great sensuality from a masculine point of view (Blake, 1994) and intended to limit mobility of women, resulting in substantial disability in basic physical activity (Greenhalgh, 1977).

Previous research interest of foot binding has widely focused on the origin, social and historical background, cultural significance, and feminist perspective. A few studies pay attention to analysis on morphological characteristics of bound feet initially reported by Jackson (1990) with a systematic description. Further studies mainly concern imaging analysis based on footprints and computed tomography (CT) images (Munk and Poon, 1996; Reischl et al., 2008; Howard and Pillinger, 2010). Three-dimensional (3D) skeleton model of bound foot has also been reconstructed by segmenting X-ray images in image processing software. Apart from the deformity of the second to fifth phalanges, the calcaneal-first metatarsal angle, talo-first metatarsal angle and horizontal metatarsal angle illustrated a broken longitudinal arch which is extremely high compared with that of a normal foot (Zhang et al., 2014). With regard to biomechanical consequences, Zhang et al. (2015) investigated gait kinematics of bound feet women and results indicated reduced range of motion in the ankle. Gu et al. (2015) compared plantar pressure between bound foot and normal foot, observing increased plantar loading on the rearfoot in bound foot.

The gross pressure distribution recorded from experimental measurement could unilaterally interpret the functional role of different anatomical components. The states of internal stress/strain in bones and soft tissues and contact pressure at joints remain unaddressed due to the difficulties and limitations of the experimental approach. Complementary to measurements, finite element (FE) analysis is capable of simulating the mechanical responses of biological systems via a numerical model, complex material properties and varying boundary and loading conditions. Many geometrically accurate 3D FE models have been developed to evaluate the biomechanics of foot-ankle complex. Using FE method, Cheung et al. (2006) reported that the increasing Achilles tendon force caused increased strain on plantar fascia. Sun et al. (2012) predicted higher the stress and strain on the plantar fascia and metatarsal in the high-arched foot compared with low-arched foot. A non-linear FE model was developed recently to assess the effect of tendon force on foot biomechanics by a force sensitivity study (Morales-Orcajo et al., 2017).

Despite of the severe deformity, some women with bound feet, especially those living in rural areas, working outdoor has been reported (Qin et al., 2015), indicating the certain remaining mechanical competence of the bound foot. An in-depth analysis on internal foot biomechanics will be of great clinic and historic significance in better understanding of injury mechanism and changes in foot function as a result of foot binding. Literature documented that cartilage is regularly subjected to high levels of stress is stiffer than that under low stress levels (Swann and Seedhom, 1993). Thus, the cartilage stiffness of bound foot is supposed to increase due to the chronic tight compression of the bandage. The purpose of this study is to investigate cartilage stiffness effect on the internal foot structure of Chinese bound feet while weight-bearing by predicting stress/strain within and between different structural components and contact pressure at joints using FE analysis.

## Materials and Methods

### Finite Element Model Construction

The geometrically accurate FE model of the bound foot was reconstructed from CT images of a 92-year old female (height: 153 cm; weight: 47.5 kg). The coronal CT images were obtained with a space interval of 2 mm without weight-bearing. The images were segmented using MIMICS 16.0 (Materialise, Leuven, Belgium) to obtain the boundaries of the skeleton and the soft tissue. The uneven surfaces caused by the stacking of the medical images were processed using Geomagic Studio 2013 (Geomagic, Inc., Research Triangle Park, NC, United States). Each surface component was then imported into Solidworks 2016 (SolidWorks Corporation, MA, United States) individually to form solid parts. Except the first to fifth phalanges were fused, the other bony components were sagittally connected with cartilaginous structures created by Boolean operations allowing relative bone movements. The encapsulated soft tissue was subtracted from the whole foot volume by the bony and cartilaginous structures. The whole foot model consisted of 28 foot bony segments, including tibia, fibula, talus, calcaneus, cuboid, navicular, three cuneiforms, five metatarsals and 14 phalanges. Link elements (tension-only) were used to simulate ligaments bearing the tension load. A total number of 72 ligaments and five plantar fascia were included and defined by connecting corresponding anatomical locations on the bones by reference to an anatomy book [13].

Mesh sensitivity was performed to ensure the accuracy and validity of the model and optimum requirement on the computational resources. The tissues of bone, cartilage and encapsulated soft tissue were meshed tetrahedral solid elements (Gu et al., 2010a,b). The ANSYS Workbench 17.0 (ANSYS, Inc., Canonsburg, United States) was used for FE analysis. Automated surface-to-surface contact algorithm in ANSYS Workbench was used to simulate the interaction between the surfaces of the cartilaginous and bony structures. The contact algorithm considers both contact elements faces which in turn prevent any penetration of the nodes on the target surface into the master surface. All the bones and cartilages were bonded to the encapsulated soft tissue.

### Material Properties

All the materials except for the soft tissue were considered isotropic and linearly elastic with material properties obtained from previous literature (Cheung et al., 2005). The material constants of Young’s modulus *E* and Poisson’s ratio ν were given to describe the elasticity. To conduct the sensitivity analysis of cartilage stiffness, the incremental Young’s modulus of 1, 5, 10, and 15 MPa were assigned to the cartilage. Young’s modulus of 1 MPa was chosen as a reference value to present the normal cartilage stiffness (Cheung et al., 2005). The encapsulated soft tissue was set as non-linear hyperelastic material which was defined as Moonley–Rivlin model (Coefficients: C10 = 0.08556; C01 = -0.05841; C20 = 0.03900; C11 = -0.02319; C02 = 0.00851; D1 = 3.65273; D2 = 0.00000) (Qiu et al., 2011). The element types and material properties used are listed in **Table 1**.

**Table 1:** Material properties and mesh element types for the foot model components.

Component	Element type	Young’s modulus E (MPa)	Poisson’s ratio *ν*	Cross-section area (mm^*2*^)
Bone	Quadratic tetrahedron	7300	0.3	-
Cartilage	Quadratic tetrahedron	1, 5, 10, 15	0.4	-
Ligaments	2-node linear 3-D spar	260	0.4	18.4
Plantar fascia	2-node linear 3D spar	350	0.4	58.6
Plate	Quadratic hexahedron	17000	0.1	-
Soft tissue	Quadratic tetrahedron	-	-	-

### Boundary and Loading Conditions

**FIGURE 1:**
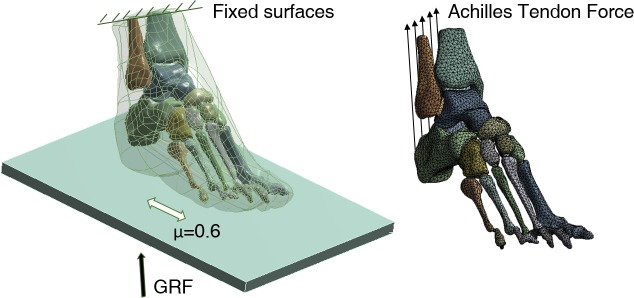
Three-dimensional finite element (FE) model and application of boundary and loading conditions. GRF, ground reaction force.

A balanced standing condition was considered for the FE analysis. The superior surfaces of the encapsulated soft tissue, distal tibia and distal fibula were fixed. The foot-ground interaction was simulated as a foot-plate system (**Figure 1**). The plate was assigned with an elastic property to simulate the concrete ground support (**Table 1**). The plate was allowed to move freely only in the vertical direction. A vertical ground reaction force (GRF) of a half-body weight (210 N) was applied at the inferior surface of the plate. The interaction between the foot plantar surface and the superior surface of the plate was simulated as contact with friction. The coefficient of friction was set to 0.6 (Cheung et al., 2005).

Only the Achilles tendon force (ATF) was considered, while other intrinsic and extrinsic muscle forces were neglected. Five equivalent force vectors representing the ATF were applied at the insertion of the posterior calcaneus (**Figure 1**). The normal ATF is commonly estimated as 50% of the force applying on the foot while balanced standing in research (Cheung et al., 2006; Chen et al., 2010) reported that the increase of ATF resulted in anterior shifting of center of pressure (COP). In order to simulate the posterior shifting of COP due to foot binding (Gu et al., 2015), the magnitude of normal ATF multiplied by a factor of 0.8, 0.5, 0.3 was also applied to Achilles tendon, respectively. **Figure 4A** shows the definition of COP ($\bar{x},\bar{y}$) location which was calculated as

$$\bar{x}=\sum p_{i}x_{i}/\sum p_{i}\text{, }\bar{y}=\sum p_{i}y_{i}/\sum p_{i}$$

Where *p_*i*_* is the nodal pressure values at each FE node (x_*i*_, y_*i*_).

### Model Validation

For the purpose of balancing reliability and time cost, the basic pressure-based validation approach is more advocated and used in the majority of studies (Behforootan et al., 2017). The FE model was validated by comparing plantar pressure distribution and peak pressure obtained from computational prediction in FE software and experimental measurement by a Novel emed pressure platform (Novel, Munich, Germany) in a standing position. The measurement was performed on the same subject who had volunteered for the medical image scanning. The participant was asked to stand still on the pressure platform for 5 s. Data of the middle 3 s were selected and averaged.

## Results

### Finite Element Model Validation

**FIGURE 2:**
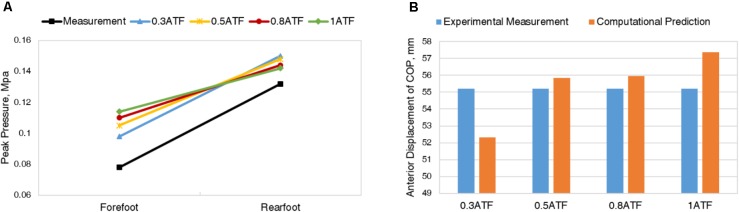
Comparison of peak pressure **(A)** and anterior displacement **(B)** of center of pressure (COP) between measurement and predictions of different Achilles tendon forces (ATFs), with Young’s modulus of cartilage, *E* = 1MPa.

**FIGURE 3:**
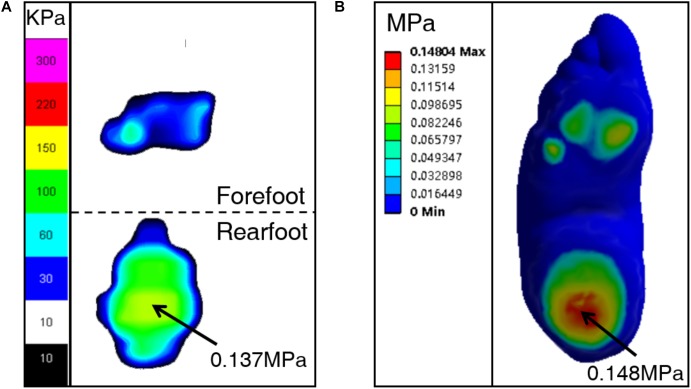
Comparison of plantar pressure distribution between measurement **(A)** and prediction **(B)**, with Young’s modulus of cartilage, *E* = 1MPa, and 50% of normal ATF.

**Figure 2A** shows the peak pressure on the forefoot and the rearfoot measured by Novel system and predicted by simulation with different ATF and *E* = 1MPa for Young’s modulus of cartilage. The values of peak pressure predicted from different ATF showed to be nuanced and were in good agreement with that tested in the *in vivo* measurement. Comparison of the anterior-posterior displacement of COP between measurement and predictions are shown in **Figure 2B**. It deviates largely with regard to 1 normal ATF and 0.3 normal ATF, while there is a better consistency between measurement and prediction for 0.5 normal ATF. Therefore, 52.5N was applied to Achilles tendon of the FE model of bound foot for further analysis. With a reference value, *E* = 1MPa for Young’s modulus of cartilage, and 0.5 normal ATF, the FE model was validated with plantar pressure measurement as shown in **Figure 3**. It shows that the plantar pressure distribution from experimental measurement and computational prediction were comparable. The peak pressure of 0.148 MPa concentrated on the heel region was predicted as compared to that of 0.137 MPa measured.

### 2-Dimensional COP Displacement

**FIGURE 4:**
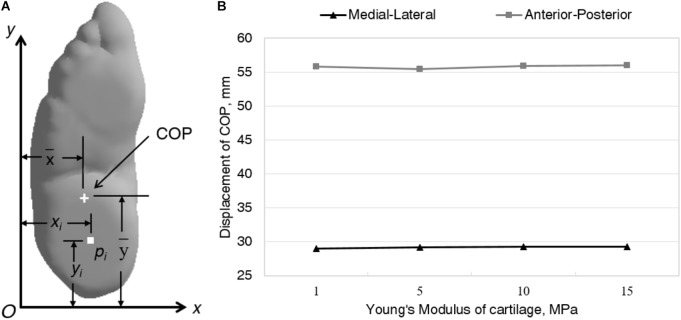
Definition of location **(A)** and comparison of 2-dimensional displacement **(B)** of COP between different Young’s modulus of cartilage.

There was a trivial effect of cartilage stiffness on the COP displacement (**Figure 4B**). In the anterior-posterior direction, it varied relatively larger than in the medial-lateral direction, but the difference is slight. The 2-dimensional displacement of (COP_x_ = 29.03 mm, COP_y_ = 55.83 mm), (COP_x_ = 29.17 mm, COP_y_ = 55.48 mm), (COP_x_ = 29.24 mm, COP_y_ = 55.9 mm), and (COP_x_ = 29.29 mm, COP_y_ = 56.05 mm) were predicted with regard to the simulated case of Young’s modulus of cartilage, *E* = 1MPa, *E* = 5 MPa, *E* = 10 MPa, *E* = 15 MPa, respectively.

### Peak Von Mises Stresses in Bones

**FIGURE 5:**
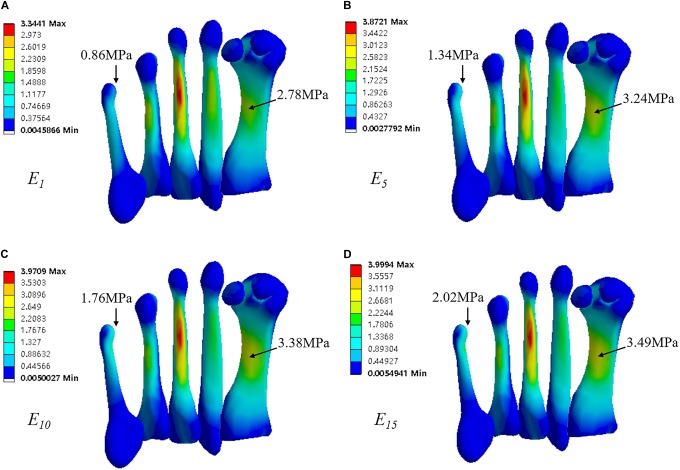
von Mises stress in five metatarsals, with Young’s modulus of cartilage, **(A)** *E* = 1MPa, **(B)** *E* = 5MPa, **(C)** *E* = 10MPa, and **(D)** *E* = 15MPa. Rectangle indicates location of peak stress in the fifth metatarsal.

**Table 2** displays the peak von Mises stresses in selected bones with incremental cartilage stiffness. In all simulated cases, the highest Mises stresses were predicted at the mid-shaft of the third metatarsal, followed by the first metatarsal (**Figure 5**). As comparing to the reference value, *E* = 1 MPa, a general increase in peak von Mises stress in bones was observed in the simulated cases of larger Young’s modulus of cartilage. It remained unchanged or minimal changes in the peak stress in the second metatarsal and calcaneus with increased cartilage Young’s modulus. Compared with *E* = 1 MPa, the peak von Mises stress increased 65.9% in navicular, 55.8% in the fifth metatarsal, 44.8% in cuboid, 16.5% in the first metatarsal, 15.9% in the third metatarsal, 11% in talus, and 2.8% in the fourth metatarsal in the simulated case of *E* = 5MPa; As the Young’s modulus increased to 10 and 15 MPa, sequential increase of stress only existed in the distal site of the fifth metatarsal and the mid-shaft of the first metatarsal, but the differences are very small (**Figure 4**).

**Table 2:** Peak von Mises stresses (Unit: MPa) in selected bones with different cartilage stiffness.

Cartilage stiffness (*E*)	M1	M2	M3	M4	M5	CAL	TAL	NAV	CUB
1-MPa	2.78	1.94	3.34	2.18	0.86	1.11	1.09	0.44	0.58
5-MPa	3.24	1.94	3.87	2.24	1.34	1.12	1.21	0.73	0.84
10-MPa	3.38	1.98	3.97	2.30	1.76	1.12	1.2	0.80	0.89
15-MPa	3.49	1.97	4.00	2.30	2.02	1.13	1.22	0.86	0.89

M1, the first metatarsal; M2, the second metatarsal; M3, the third metatarsal; M4, the fourth metatarsal; M5, the fifth metatarsal; CAL, calcaneus; TAL, talus; NAV, navicular; CUB, cuboid.

### Plantar Fascia Tension and Strain

**Table 3** displays the predicted plantar fascia tension and strain with different cartilage stiffness. The plantar fascia experienced reduced total tension with the increased Young’s modulus of cartilage. The first and fourth ray sustained major tension loading and strain in all simulated cases. They underwent about 60% overall tension. The fifth ray played a minimum role assuming less than 11% overall tension. There was a slight decrease of plantar pressure strain in the fourth ray as the Young’s modulus of cartilage increased, while the changes in other rays were trivial.

**Table 3:** Plantar fascia tension and strain with different cartilage stiffness.

Plantar fascia	Tension (*N*)				Strain (%)			
	1-MPa	5-MPa	10-MPa	15-MPa	1-MPa	5-MPa	10-MPa	15-MPa
1st ray	8.83	9.07	9.03	9.00	0.043	0.044	0.044	0.044
2nd ray	4.71	4.58	4.58	4.63	0.023	0.022	0.022	0.023
3rd ray	3.67	4.06	4.13	4.18	0.018	0.020	0.020	0.020
4th ray	9.37	8.83	8.22	7.73	0.046	0.043	0.040	0.038
5th ray	3.06	2.61	2.73	2.84	0.015	0.013	0.013	0.014

### Contact Pressure of Midfoot and Forefoot Joints

**FIGURE 6:**
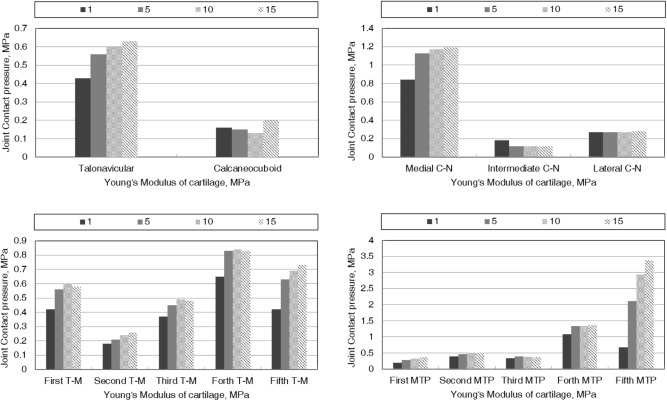
The effect of cartilage stiffness on the contact pressure of joints at midfoot and forefoot. C-N, cuneonavicular joint; T-M, tarsometatarsal joint; MTP, metatarsophalangeal joint.

**Figure 6** shows the effect of cartilage stiffness on the contact pressure of joints at the midfoot and forefoot. There was a general increase of contact pressure at mid- and forefoot joints with the increase Young’s modulus of cartilage. The increased cartilage stiffness produced obvious increase in joint contact pressure when the Young’s modulus increasing from 1 to 5 MPa. At the midfoot, joint contact pressure on the medial side (talonavicular and medial cuneonavicular joints) was larger than that on the lateral side (calcaneocuboid, intermediate cuneonavicular, and lateral cuneonavicular joints). At the forefoot, joint contact pressure at the fourth and fifth tarsometatarsal joint and metatarsophalangeal joint (MTP) was larger than others. Reduced contact pressure due to the stiffer cartilage was found at the intermediate cuneonavicular joint.

## Discussion

The practice of foot binding spanned over 1000 years in Chinese history. It began in Southern Tang Dynasty, flourished in Song Dynasty, and finally was forbidden in the early 20th century (Qin et al., 2015). To date, there are still elder women with bound feet living in some rural and impoverished areas of China, but only few. Due to exposure to chronic intense pressure of the bandage, the property of articular cartilage may vary from normal state. This study explored the effect of cartilage stiffness on biomechanical consequences of bound foot during balanced standing.

Many studies have constructed FE models from normal feet or a pathological model modified from the normal one to investigate the internal biomechanical features of the foot with a great success (Wang et al., 2016). This study developed a complete foot FE model from an elder woman with bound foot. For a foot FE model, except for the bone tissue modeled in almost all relevant studies, there is a large percent studies included ligaments, tendons, and soft tissue (Behforootan et al., 2017). About half of the existed research involved cartilages in modeling FE foot model (Behforootan et al., 2017). For studies excluded cartilaginous components, they used frictionless contact elements allowing free movements between bones. Alternatively, as performed in the model of this study, it is possible to connect two adjacent bones with low stiffness elements, which provides more realistic simulation of articular behavior. As reported in previous study, the longitude displacement of COP deviated posteriorly for bound feet in comparison with normal feet. Consistently, the COP measured in the current study located at the heel region while balance standing. Base on the prediction from a FE foot model constructed by Cheung et al. (2006) that increasing Achilles tendon loading caused anterior shifting of COP in a standing position, this study attempted to simulate the posteriorly shifted COP of bound foot by decreasing the ATF. A factor of 0.8, 0.5, and 0.3 of normal ATF was included for simulation, and the predicted results showed that the factor of 0.5 achieved the most agreeable displacement of COP between prediction and measurement. The model was validated by plantar pressure measurement and the predicted plantar pressure distribution and COP location showed a good agreement with those measured by *in vivo* experiment.

From the FE predictions, the highest von Mises stress in bones was found concentrating at the mid-shaft of the third metatarsal. FE analysis of a normal foot model indicated similar location of the highest stress, however, the magnitude is about twofold greater than that predicted in the bound foot model (Cheung et al., 2005). Moreover, according to Cheung et al. (2005), the peak von Mises stress in calcaneus of the normal foot is more than threefold greater than that predicted in the bound foot model. Normally, the calcaneus plays an important role in weight bearing, supporting about 40% of the body weight (Rodgers, 1988). Due to the severe deformity at forefoot, the heel region of bound feet experienced increased plantar pressure. Higher plantar pressure in normal foot was regarded to be associated with increased von Mises stress in bones (Chen et al., 2001). Different from what was expected, the magnitude of stress in calcaneus of the bound foot showed to be lower than the stress in the first to fourth metatarsals. This may be caused by the thickening of the fat pad at the heel. With a fivefold increase of Young’s modulus of cartilage, the stiffer cartilage affected the peak von Mises stress in most bones except for the second metatarsal and calcaneus, indicating higher risk of foot pain even after foot binding process complete. In fact, apart from the initial pain in the 1st year of foot binding, long-term pain was also complained (Stone, 2012). As the Young’s modulus increased to 10 and 15 MPa, the first and fifth metatarsal showed minor increments of stress, indicating that these are possible areas are more vulnerable to pain as cartilages persistently stiffening during the lifetime.

The FE analysis showed reduced total tension force with the increase of Young’s modulus of cartilage. For the incremental Young’s modulus, the five rays of plantar fascia segments sustained 14.82, 14.58, 14.35, and 14.19% of the applied force on the plate, respectively, which is prominently less than the prediction (44% of applied force) from a normal foot model (Cheung et al., 2006). Regarding to the strain, all five segments decreased by one order of magnitude as to bound foot compared to that predicted from a normal foot (Yu et al., 2008). The reduced plantar fascia tension and strain demonstrated the broken structure of longitude foot arch. One FE study evidenced that the high-arched foot generated higher plantar fascia stress and strain than the normal foot (Sun et al., 2012). The current study predicted adverse result in bound foot, demonstrating that the extremely high arch is very likely to resulting in function damage in supporting the foot shape. Admittedly, the bound foot is associated with resembling the state of high-heeled shoes. Yu et al. (2016) found decreased total tension and average strain of plantar fascia when the heel height increased from 0 to 5.08 cm, however, this is still greater than the predictions from bound foot. Furthermore, the effect of cartilage stiffness on the plantar fascia strain was minimal. The change of strain in the fourth ray showed to be relatively noticeable with a gradual decrease as the Young’s modulus of cartilage increased from the reference value to 15-fold. In a study evaluating the calcaneus functional adaptation in foot binding, the mechanical influence of plantar fascia was even disregarded (Reznikov et al., 2017).

The predicted contact pressure at midfoot and forefoot joints showed a general increase with the incremental Young’s modulus of cartilage. Similar variation was observed in joint contact pressure with respect to the restricted foot due to ankle arthrodesis. Wang et al. (2015) presented larger contact pressure at midfoot joint in the foot with ankle arthrodesis than a normal one. Despite the severe deformity of the fourth and fifth phalanges, the structure of MTP at these sites practically remains intact. The contact pressure at the fourth and fifth MTP exhibited higher than other MTP, which may result from the hyperflexed position of the phalanges. At the midfoot, the medial cuneonavicular joint showed prominent contact pressure. As demonstrated previously, the excessive contact stress on articular surface is thought to be a predominant association with osteoarthritis (Buckwalter and Martin, 2006). The medial side of midfoot is considered an area of high risk of arthritis. Effect of cartilage stiffness on contact pressure was most pronounced in the fifth MTP. It can be speculated that the increased contact pressure at the fifth MTP potentially aggravates the pain caused by foot binding.

It should be noted that the effect of cartilage stiffening considered in this study was simplified by a uniform increase of Young’s modulus over all cartilages of the entire foot. In the real cases, the degree of cartilage stiffening may vary at different foot segments. Lacking specific information about mechanical properties of plantar fascia for bound foot, this study assumed the Young’s Modulus, Poisson’s Ratio, and cross-section area the same as those of the normal foot. Since the Achilles Tendon plays a more important role while balanced standing, other intrinsic and extrinsic muscle forces were neglected. Based on validated plantar pressure distribution and COP location, the resulted predictions are able to represent the tendency of mechanical responses to stiffened cartilages. In addition, the foot binding was usually performed based on the experience of one’s mother (Stone, 2012) with the main purpose of restricting foot size. An extremely high arch and folded phalanges would be formed commonly, while the relative rearrangement of different phalangeal segments may vary uniquely (e.g., fully folded phalanges and semi-folded phalanges). Knowledge of this study accounts for biomechanics of bound foot that is featured by severely damaged fourth and fifth phalanges.

## Conclusion

This study constructed a subject-specific FE model of Chinese bound foot and preliminarily showed its mechanical response to stiffened articular cartilages. Compared with previous biomechanical analysis that aimed to inform the external loading (i.e., plantar pressure) of bound feet, the numerical model developed an approach for predicting changes in internal tissue stress/strain due to the abnormal foot structure, which will therefore improve the comprehension of the theories of bound foot injury and function loss. More importantly, simulations on the effect of increased cartilage stiffness provided additional interpretation on the mechanism of aggravating foot pain and functional damage of longitude arch associated with foot binding. We found that the peak von Mises stress in the fifth metatarsal increased obviously as Young’s modulus of cartilage increased. Cartilage stiffening also caused generally increased joint contact pressure at medial mid-foot and lateral forefoot. Larger bony stress and joint loading are the potentials for rising pain. The reduced total tension of plantar fascia with stiffer cartilage may indicate the progressively weakened weight-bearing ability of the longitude arch. Further work is supposed to analyzing higher loading condition of stance phase in gait.

## Ethics Statement

This study was approved by the Ethics Committee of the University of Ningbo (ARGH20160818). Participant was informed of the purpose, requirements and process of this experiment.

## Author Contributions

YZ and YG conceived and designed the experiments and performed the experiments. YZ, JA, JB, and YG analyzed the data, interpreted results of research, wrote the paper and involved in the editing process of the manuscript.

## Conflict of Interest Statement

The authors declare that the research was conducted in the absence of any commercial or financial relationships that could be construed as a potential conflict of interest.
